# Parameters influencing hand grip strength measured with the manugraphy system

**DOI:** 10.1186/s12891-018-1971-4

**Published:** 2018-02-14

**Authors:** Alice Wichelhaus, Christoph Harms, Julia Neumann, Steffen Ziegler, Günther Kundt, Karl Josef Prommersberger, Thomas Mittlmeier, Marion Mühldorfer-Fodor

**Affiliations:** 1 Clinic for Trauma, Hand and Reconstructive Surgery, University of Rostock Medical School, Schillingallee 35, 18057 Rostock, Germany; 2 Clinic for Hand Surgery, Rhön Klinikum AG, Salzburger Leite 1, 97616 Bad Neustadt an der Saale, Germany; 3 Institute for Biostatistics and Informatics in Medicine and Ageing Research, University of Rostock, Ernst-Heydemann Str. 8, 18057 Rostock, Germany

**Keywords:** Grip strength, Hand length, Cylinder grip, Manugraphy system, Grip size

## Abstract

**Background:**

This study aimed to determine whether sex, hand length and the individual training status affect hand strength and whether these measurements differ if they are recorded using the Jamar dynamometer or a new cylindrical measuring system.

**Methods:**

For this purpose, 152 healthy adults were examined using a new manugraphy measuring system (novel, Munich, Germany) comprising two measuring cylinders of different sizes and a Jamar electronic dynamometer with two grip positions corresponding approximately to the sizes of the cylinders. A descriptive analysis was performed as well as a correlation analysis using the Pearson correlation coefficient. To prepare predictive models, multiple linear regression analyses were carried out to determine factors that influence the force and *p* ≤ 0.05 was considered statistically significant.

**Results:**

A significant difference in the maximum and mean strength was observed that is dependent on sex, with men stronger than women, in line with expectations, and hand length, with small hands able to exert less force than large hands. No consistent increase in strength could be attributed to repetitive manual loads applied either at work or in leisure activities.

**Conclusions:**

Both measurement techniques yielded similar results, suggesting that manugraphy is well suited for clinical research purposes because it not only takes measurements that are just as reproducible and valid as the conventional measurement technique but in doing so measures not just the total strength of a hand but also enables more precise comparisons of isolated hand regions applying dynamic measurements.

## Background

To be able to estimate changes in hand function after injuries, over the course of healing or as part of an expert examination, objective measurement techniques are required, ones that also allow classification of the results of scientific studies when comparing different treatment options. The loss of strength in the hand with the power grip is an important reduction in the overall functionality of a hand [1]. Measuring hand strength is also used as a simple method to assess general muscle strength to identify functional deficits [2]. There are indications that mortality and morbidity is higher for major procedures on the gastrointestinal tract or after arthroplasty in patients with less hand strength before surgery [3, 4]. The device used to measure hand strength most commonly cited in the literature is the Jamar dynamometer, which seems to be accepted as the gold standard against which other strength measuring devices are compared [5]. The reliability (r > 0.98) and validity (v > 0.95) of the Jamar dynamometer can be considered high [5–7]. The Jamar dynamometer is small and portable but at 0.7 kg (kg) it is quite heavy. Very weak individuals may therefore have problems holding the device without assistance. There is a scale on which the hand strength can be read in 1 kg or 2.2 pound steps. A force of at least 3–4 pounds/1.3–1.8 kg must be exerted to deflect the indicator needle. The reading error increases as the force decreases [8]. It is not possible to allocate the force exerted to isolated regions of the hand. Ergonomic studies to determine the contact forces between a tool and the palm often use gloves with pressure recording sensors or sensors embedded in an elastic mat that are placed around the device to be tested [9–13], so that the cylinder grip that is important in routine use can be imitated. The manugraphy system (novel biomechanics laboratory, Munich, Germany) also works with cylinders that are enclosed in pressure recording mats. This allows highly accurate measurement data to be recorded. Unlike the Jamar dynamometer, the manugraphy system can determine how much force is exerted by different hand regions and fingers during the gripping process [1]. This may enable the complex gripping process to be evaluated with greater precision, even for patients with functional restrictions after a hand injury or as a result of nerve damage, for example. Most protocols dealing with grip measurement with cylindrical handles are ergonomic studies that investigate optimized designs to reduce physical effort and the risk of musculoskeletal disorders for handles used across all industrial sectors [9–12]. The manugraphy system can add to the understanding of time flow and force distribution during power grip.

Before using the device clinically, the physiological parameters that influence grip strength need to be determined. Previous studies using the manugraphy system have confirmed that age does not correlate with hand strength [1] and that the strength distribution pattern does not fundamentally differ between the dominant and non-dominant hand of an individual [14]. Handedness itself does not have a consistent effect on grip strength [1, 14].

### Objective of the study

The aim of the study was to determine whether sex, hand length and repetitive loading of the hands at work or during leisure activities, that is the training status, affect hand strength and whether these measurements differ if they are recorded using the Jamar dynamometer or the manugraphy system.

## Methods

### Participants

In this 2-centre study, 152 healthy subjects were recruited in two hospital setting, one localized at the Baltic Sea in north-eastern Germany, the other in a rural district located in central Germany. We studied a population of healthy male and female subjects working as members of the medical and sports science departments of the faculty as well as construction workers, office employees and students who participated voluntarily without payment. Exclusion criteria were a history of injuries or existing diseases of the upper extremities, the presence of myofascial syndromes, rheumatic disease, multiple sclerosis or malignant underlying diseases. The subjects’ handedness and the length of both hands in centimetres (cm) were recorded. The distance between the wrist fold and the tip of the middle finger with the hand held straight and stiff was measured in centimetres (cm). Hands with a length ≤ 17.5 cm were defined as small while hand lengths of 17.5 cm to ≤19 cm were classified as medium and hand lengths > 19 cm were defined as large.

The manual training status was ranked by informations about manual loading at work and during leisure activities.The participants subjectively grouped themselves into the following four categories:

1. activities without special manual loading, no sports at all
2. activities with changing manual loading, sports without manual loading
3. activities with constantly repeated manual loading without greater force expenditure, sports with manual loading
4. activities with constantly repeated manual loading with force expenditure, sports with strong manual loading.

### Device-based technology

#### The manugraphy system

The novel® manugraphy system (novel biomechanics laboratory, Munich, Germany) is available with different sized cylinders that are enclosed in soft elastic pressure recording mats. Two calibrated pressure sensors per square centimetre are embedded in the mat. Each sensor element is 7.07 × 7.07mm^2^ (millimetre) in size. For the current test series, two different cylinder sizes were used, the smaller measuring 150 mm in circumference with a diameter of 48 mm, the bigger measuring 200 mm circumference and a diameter of 64 mm. The 150-mm cylinder records signals from 672 sensors and the 200-mm cylinder records signals from 896 sensors. Each sensor is calibrated to a maximum pressure of 600 kPa with a measurement error of < 5%. The sensors transmit their signals with a frequency of 20 Hz to the manugraphy analysis computer that is wired to the device. During the measurement the dynamics of the force distribution of the hand are displayed on a monitor. The force component vertical to the surface is calculated by the software separately for each sensor so that the force distribution across the different areas of the hand can be determined. The sum across all sensors gives the overall force, which is displayed as a force over time-diagram in newton (N). The force exerted on each individual sensor is colour coded and reported as a numerical value. The sensitivity threshold was set at 5 kPa so that the threshold per sensor is 0.25 N. During the measurement, the software determines the average force applied to the cylinder surface occupied by the examined hand. The measured pressure values of all those pressure sensors to which more pressure is applied than the previously calibrated air pressure is summed up. For better visualization a dynamic representation of the measuring foil in the form of a handprint can be deducted. The reliability of the manugraphy sytem is high with r > 0,97 [1, 14].

#### The biometrics measuring device

The G200 Biometrics measuring device (Biometrics Ltd., Newport, Great Britain) is a modified digital Jamar dynamometer with a reliability of r > 0,98 [5–7]. The classic Jamar dynamometer is made up of two handles that are drawn towards one another with no perceptible deflection of the grip. An incompressible oil column absorbs the force exerted and transmits it. The handles can be adjusted to 5 different lock positions. Handle positions 3 and 4 are comparable to the measuring cylinder diameters of 4.8 and 6.4 cm of the manugraphy measuring cylinder which is why these two handle positions were selected for the study. The force exerted is recorded electronically by a pressure sensor and the analysis is carried out on a personel computer (PC). This allows the force to be recorded dynamically over time. Software automatically determines the maximum force and the mean force whereas the Jamar values, measured in kilogram, are converted to newton (9.8 *N* = 1 kg × 9.81 m/s^2^) so that the values can be compared to the manugraphy data. The Jamar dynamometer has been shown to produce measurements with a high intra-rater and inter-rater reliability. Manual reading errors are avoided by the use of the computer connected tool [6–8].

### Test protocol

The individuals were examined by one investigator per centre using a standardised protocol. Prior to inclusion in the study a written declaration of informed consent was obtained from the study participants after the issues and risks associated with participating in the test were explained. The study protocol corresponds to the specifications in the Declaration of Helsinki and was checked and approved prior to the start of the study by the relevant ethics committee.

Each participant was examined over three weeks on three different days with at least 24 h between each appointment. Half the tests were started with the measurement using the manugraphy system and the other half were started with the Jamar dynamometer and the order was changed over for each participant at the next visit. The initial choice was determined randomly.

To minimise any influence due to the investigator, throughout both measurement procedures the subjects were accompanied by an automated voice that told the subjects to compress the measuring device for 5 s with maximum strength and then to rest for 10 s without releasing the device from the hand or changing the position of the hand on the device. This sequence could be transferred to a clinical setting as it is neither too time consuming nor too complicated. Three measurements were carried out with each device at every visit, starting with the left hand and then the right hand. Based on the recommendations of the American Society of Hand Therapists, the test participants sat on a stool without a back or arm rests with the upper arm resting on the upper body and with the shoulder in a neutral position and the elbow held at an angle of 90° [15]. The wrist position could be freely chosen by each participant assuming that a position would be selected that allowed the individual to apply the greatest force. The seating and joint positions throughout the entire measurement were recorded on video so that any influences in this regard would be apparent.

The subjects were not able to see the recording of the force curve on the PC monitor during the test to exclude any possible influence [16]. The measurements were started with the smallest handle position and the smaller cylinder for both hands and continued with the larger handle position and the larger cylinder. For both measuring methods, the maximum force during the individual measurement and the mean force were recorded. The mean force was calculated from the interval of the middle 3 s of the 5-s exertion phase to avoid the initial delay in the muscle tension after the command is given as well as the anticipatory relaxation at the end of the loading phase [12].

### Statistical methods

All data were saved and analysed using the Windows® based statistical software package for the social sciences SPSS (IBM, Armonk, USA). In the first step, a descriptive analysis was carried out. The quantitative characteristics were described using the mean ($\overline{x}$), standard deviation (SD), minimum (min), maximum (max) and the number of observations (n) available. For the qualitative characteristics, the absolute frequency and percentage frequency were stated for the individual characteristic. The relationship between the values obtained with the two measurement techniques was quantified in correlation analyses using the Pearson correlation coefficient as the measure of association. To prepare predictive models, multiple linear regression analyses were carried out for the two methods to determine factors that influence the force/power. In a stepwise process successive to this, a model was determined that best explained the desired relationship and avoided those parameters that contained redundant information about the γ.

All *p* values are the result of two-sided statistical tests and *p* ≤ 0.05 is considered statistically significant as a general principle.

## Results

All study participants were of working age between 18 and 65 years with a mean age of 35.8 years (SD 11). As requested by the test-protocol there was a balanced sex distribution (76 women, 76 men). The hands were classified as small in 53 (34.9%), medium in 53 (34.9%) and large in 46 (30.3%) of the subjects examined. For male participants average hand length accounted for 18.6 cm in comparison to an average hand length of 17.0 cm for the female participants.

For each measuring system the maximum force and the mean force of the dominant and non-dominant hand were compared for both measuring steps (small handle and large handle, small and large grip position, respectively. This yields 8 parameters per measuring device for each potential influential factor.

### Influence of the parameter ‘sex’

**Fig. 1:**
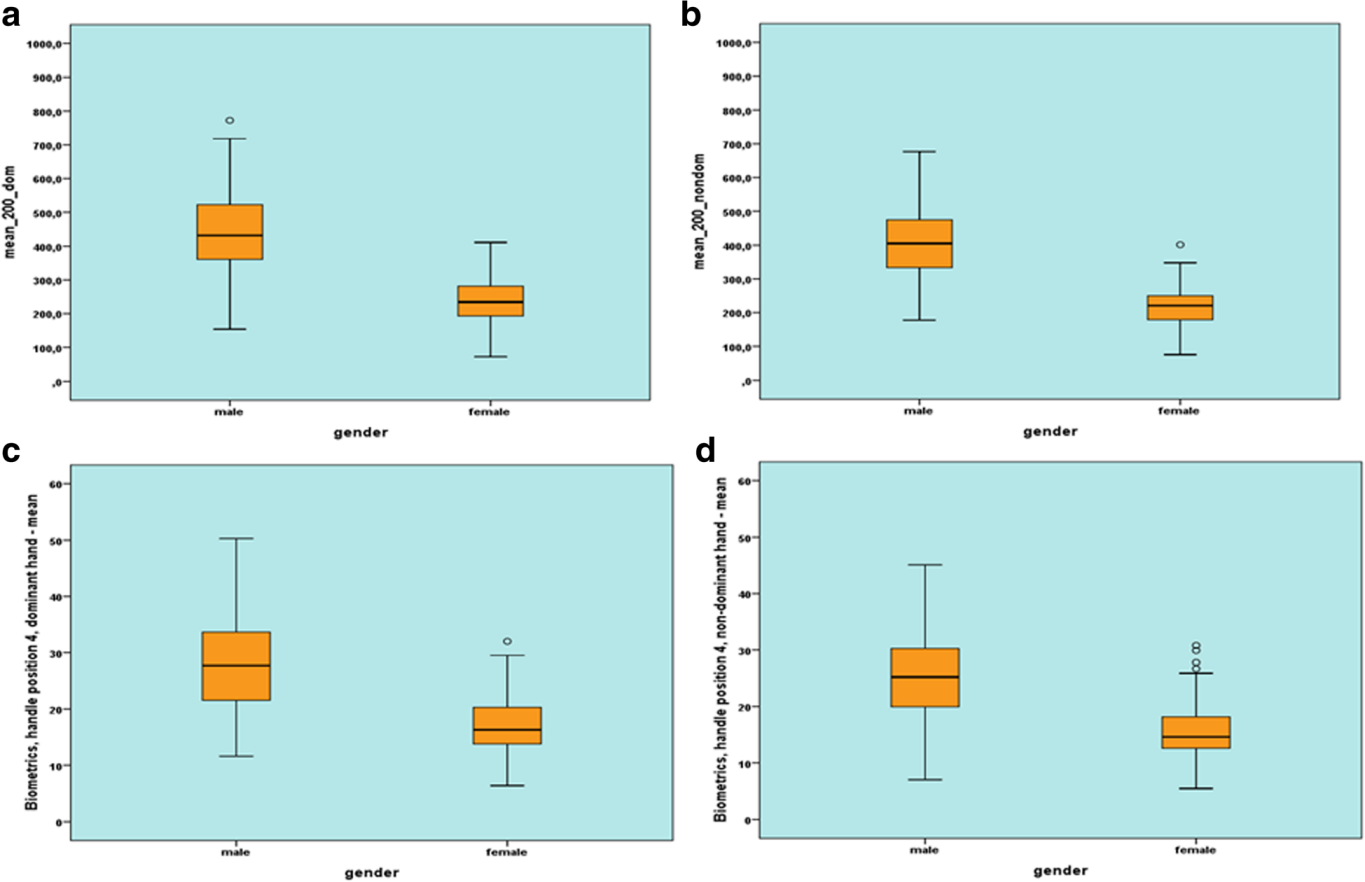
Box plot and whiskers diagram of the mean strength values for women and men. **a**: Measurement of mean strength values for the dominant hand with the 200-mm manugraphy measuring cylinder. **b**: Measurement of mean strength values for the non-dominant hand with the 200-mm manugraphy measuring cylinder. **c**: Measurement of mean strength values for the dominant hand with Biometrics handle position 4. **d**: Measurement of mean strength values for the non-dominant hand with Biometrics handle position 4

When measuring with the manugraphy system, significant differences were observed for the small and the large cylinder between the two sexes for all 8 parameters. The 76 men examined exerted significantly larger maximum and mean forces with both their dominant and non-dominant hand compared to the 76 women examined. Similar results were obtained for the Biometrics system (Table 1, Fig. 1a–d).

**Table 1:** Comparison of the force values attained by male and female participants with the manugraphy and Biometrics-system. For each participant the maximum and the mean grip force were calculated regarding both the dominant and the non- dominant hand

*Measuring device*	*Parameter*	N men	Resultant force (N)	SD	N women	Resultant force (N)	SD	p
manugraphy	maximum force, 150 mm cylinder, dominant hand	76	612.89	156.14	76	348.84	89.09	< 0.001
	mean force, 150 mm cylinder, dominant hand	76	558.76	153.24	76	307.81	85.87	< 0.001
	maximum force, 150 mm cylinder, non-dominant hand	76	580.53	147.56	76	327.07	82.65	< 0.001
	mean force, 150 mm cylinder, non-dominant hand	76	527.29	143.88	76	288.39	81.23	< 0.001
	maximum force, 200 mm cylinder, dominant hand	76	490.67	123.11	76	274.04	64.41	< 0.001
	mean force, 200 mm cylinder, dominant hand	76	443.21	121.79	76	239.23	63.75	< 0.001
	maximum force, 200 mm cylinder, non-dominant hand	76	458.58	109.94	76	252.33	59.69	< 0.001
	mean force, 200 mm cylinder, non-dominant hand	76	411.22	109.00	76	219.03	59.62	< 0.001
Biometrics	maximum force, handle position 3, dominant hand	76	342.66	85.54	76	226.80	62.29	< 0.001
	mean force, handle position 3, dominant hand	76	307.45	89.07	76	196.59	59.55	< 0.001
	maximum force, handle position 3, non- dominant hand	76	317.06	81.23	76	213.36	61.99	< 0.001
	mean force, handle position 3, non-dominant hand	76	282.72	81.23	76	185.51	59.45	< 0.001
	maximum force, handle position 4, dominant hand	76	301.56	79.85	76	197.67	56.02	< 0.001
	mean force, handle position 4, dominant hand	76	270.66	81.52	76	172.17	53.17	< 0.001
	maximum force, handle position 4, non-dominant hand	76	277.82	73.18	76	180.60	53.86	< 0.001
	mean force, handle position 4, non-dominant hand	76	247.31	73.28	76	155.68	50.33	< 0.001

### Influence of the parameter ‘hand length’

Hand length proved to have a significant influence on the hand strength.

**Fig. 2:**
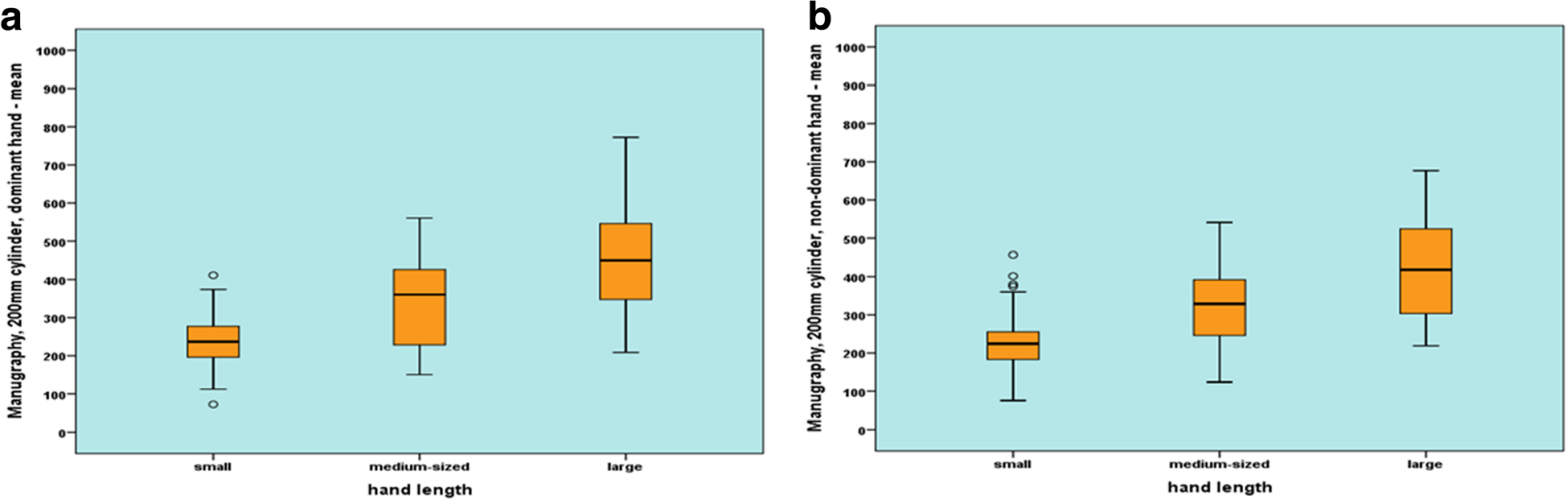
Box plot and whiskers diagram of the mean strength values for small, medium and large hands. **a**: Measurement of mean strength values for the dominant hand with the 200-mm manugraphy measuring cylinder. **b**: Measurement of mean strength values for the dominant hand with Biometrics system, handle position 4

With the manugraphy system, the strength values for the 8 parameters increased from 26.3 to 74.5 N per centimetre of increase in hand length. For the Biometrics system, strength increased from 14.32 to 17.27 N when hand length increased by one centimetre (Table 2, Fig. 2a+b).

**Table 2:** Comparison of force values attained by participants with small, medium-sized and large hands measured with the manugraphy and Biometrics-system. For each participant maximum and mean grip force were calculated regarding the dominant and the non- dominant hand

*Measuring device*	*Parameter*	N Hand length ≤17.5	Resultant force (N)	SD	N Hand length 17.5- ≤ 19 cm	Resultant force (N)	SD	N Hand length > 19 cm	Resultant force (N)	SD	p
manugraphy	maximum force, 150 mm cylinder, dominant hand	53	329.04	79.06	53	484.87	134.44	46	651.17	166.78	< 0.001
	mean force, 150 mm cylinder, dominant hand	53	291.04	78.62	53	439.68	132.68	46	589.81	167.92	< 0.001
	maximum force, 150 mm cylinder, non-dominant hand	53	322.66	95.40	53	460.77	120.14	46	631.93	168.14	< 0.001
	mean force, 150 mm cylinder, non- dominant hand	53	286.69	93.03	53	414.93	119.37	46	571.44	168.09	< 0.001
	maximum force, 200 mm cylinder, dominant hand	53	273.69	65.55	53	383.78	118.03	46	505.90	145.70	< 0.001
	mean force, 150 mm cylinder, dominant hand	53	240.09	66.29	53	342.52	117.33	46	456.24	141.69	< 0.001
	maximum force, 200 mm cylinder, non-dominant hand	53	265.85	77.45	53	358.28	111.38	46	479.99	136.76	< 0.001
Biometrics	mean force, 200 mm cylinder, non-dominant hand	53	233.08	75.96	53	317.56	108.56	46	429.38	135.48	< 0.001
	maximum force, handle position 3, dominant hand	53	236.62	72.20	53	283.17	88.19	46	341.98	94.08	< 0.001
	mean force, handle position 3, dominant hand	53	206.79	68.47	53	250.06	88.88	46	306.27	97.71	< 0.001
	maximum force, handle position 3, non-dominant hand	53	227.19	74.16	53	260.55	82.99	46	326.57	85.05	< 0.001
	mean force, handle position 3, non-dominant hand	53	198.75	70.93	53	229.36	80.34	46	291.65	85.94	< 0.001
	maximum force, handle position 4, dominant hand	53	204.24	64.75	53	245.54	77.59	46	306.56	86.13	< 0.001
	mean force, handle position 4, dominant hand	53	178.25	60.63	53	218.96	77.20	46	286.75	88.47	< 0.001
	maximum force, handle position 4, non-dominant hand	53	192.08	65.24	53	226.22	75.05	46	286.74	76.52	< 0.001
	mean force, handle position 4, non-dominant hand	53	167.85	61.51	53	199.34	73.48	46	252.99	78.68	< 0.001

The differences between small and large hands amounted to 45–51% with the manugraphy system, whereas the participants with large hands exerted grip forces greater by 30–36% than the individuals with small hands measured with the Biometrics system. Additionally, the correlation coefficients showed that the hand length has a greater influence for the manugraphy system than for the Biometrics system.

### Influence of the parameter ‘manual loading’

**Fig. 3:**
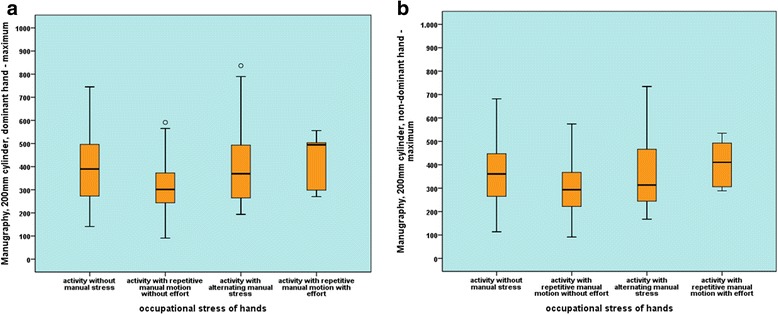
Box plot and whiskers diagram of the maximum strength for activities without manual loading, repetitive activities without a large applied force, activities with variable manual loading and repetitive activities with applied force. **a**: Measurement of the maximum strength of the dominant hand with the manugraphy system, 200-mm cylinder. **b**: Measurement of the maximum strength of the dominant hand with Biometrics system, handle position 4

The participants were asked to subjectively assess their training status regarding occupational and leasure activities. According to the self-assessment, they were assigned to a specific group with 4 different training states. A significant difference between the maximum and mean strengths could not be confirmed for any of the 8 parameters neither for the manugraphy system nor the Biometrics system between the 4 groups when considering different manual loading of the hands (Fig. 3).

A statistically significant difference between the dominant and non-dominant hand could not be determined depending on the training status.

Differences between the two study centers could not be observed proving the confirmability and stability of the measuring devices [1, 14]. In this study we demonstrated that the manugraphy system measures just as accurately and reproducibly as a Jamar dynamometer and the correlation between the measurements recorded by both devices is high [14].

## Discussion

Measuring grip strength with the Jamar dynamometer is an established and valid method that is often used in studies and routine clinical practice. It is easy to use and reproducible results are achieved [1, 5–7, 14]. However, users must be aware that the Jamar dynamometer allows a unidirectional force measurement only. Therefore some of the forces exerted when gripping are not recorded, particularly forces that are transmitted through the fingertips or the distal phalanx of the thumb [17]. The measuring cylinders of the manugraphy system solve this problem. All forces applied vertically to the sensor mat can be measured. Shear forces are disregarded in the process [11, 18]. Overall, higher forces were measured with the manugraphy system than with the Jamar dynamometer. This is due to the fact that there is a summed multidirectional measurement across all parts of the hand on the sensor mat [1]. A high correlation between the two measurement devices was confirmed for both the maximum force and the mean force measurements [1, 14]. However, the two measurement devices have a completely different shape, surface finish and weight and are made from different materials, all of which affect the strength measurement. Physical factors such as gravity, friction and torque also play a role [19–22]. Physiological factors such as joint position and the pre-tension of the muscles and tendons also affect the result and depend on the handle shape and position [23, 24]. Generally, the wrist is held in an extension position when exerting force because this allows greater maximum forces to be obtained with the grip [25, 26]. The wrist position could be freely selected by the subjects during the measurements so it can be assumed that each subject would adopt the most favourable position. By the strict test protocol, an attempt was made to minimize other influential factors.For any planned clinical use of the manugraphy system, it may also be beneficial that the measuring cylinder is enclosed in soft and therefore comfortable sensor mats so that any pain resulting from surgical scars may have less of an effect when gripping than the hard metal handles of a Jamar dynamometer [27].

In accordance with existing literature, this study verified that sex has an influence on hand strength [28–30]. It is interesting that the strength difference between the sexes is more pronounced for the non-dominant hand than for the dominant hand. It could be speculated whether men use their non-dominant hand more often in routine work situations, so that the non-dominant hand could be in a better training status, whereas women tend to use the dominant hand only. According to Agnew et al. it has been shown that hand function is related to age and sex, men performing better regarding grip strength and “moving large objects” [31]. The increased ability to move light or heavy large objects can be explained by the general increase in hand size when comparing men to women. For clinical practice it is frequently discussed, whether it is sensible to work uniformly with a certain handle size for better comparability between study groups or if it is more valuable to use handle sizes that are individually adjusted to the length of the hand, in order to achieve the best possible results. Kong et al. showed that the optimal cylindrical handle diameter is 19.7% of the user’s hand length [32]. Basically grip force decreases when the object is relatively too big compared to the hand size due to unfavourable muscle preloading and angling of joints. To study both options, two different cylinder sizes and handle positions were used in this study.

Hand strength is clearly dependent on hand length and this also varies between the sexes. Men generally have larger hands [28, 29, 32]. A larger hand means a greater hand area is applied to the sensor mat with the manugraphy measurement system and thus stimulates more sensors when gripping than a smaller hand [1, 29, 33]. For a small hand, the large measuring cylinder or the wide grip position is more uncomfortable. Greater force must be exerted by the distal phalanges of the fingers which reduces the overall force applied [1, 34]. The hand length correlates with the height and weight of the person examined. For people of normal weight, an increase in the height and weight means an increase in muscle mass which explains the greater hand strength [28, 29, 35]. The difference in the strength measured with a hand that is one centimetre longer is more pronounced for the dominant hand than for the non-dominant hand. This difference could again be explained by routine training.

What speaks against this hypothesis is that in this test series the training status had no significant influence on the values of maximum and mean strengths. Repetitive manual loading at work or during leisure activities did not lead to significantly greater hand strength. This does not contradict the assumption that not using a hand leads to a reduction in strength [8, 33, 34]. A clear limitation is that the amount of manual loading at work and during leisure activities was raised as a purely subjective assessment by the participants themselves. A scientifically valid survey of the training status did not take place. Stunningly, the manual loading was estimated with considerable variation by the subjects even if they were employed in the same job or practicing the same sport. No data was collected about the duration and frequency of the training, meaning that the statement has very limited validity.

The differences in mean and maximum resultant forces between small and large hands were increased when measured with the manugraphy system. Partial explanation for this finding is that the finger tips do not touch the Jamar dynamometer during grip and therefore cannot take part in load transmission. Overall the values obtained with the manugraphy system were 45–100% higher than those measured with the Biometrics system, indicating that the manugraphy system provides higher sensitivity so that the exertion of the test participant during cylindrical grip is reflected more precisely.

Strength of the study is that the test-retest reliability is even higher than indicated in the literature. This speaks for the precision of the measurement technology and the constant measurement circumstances provided by an accurate test-protocol. The manugraphy system is much more complicated and time-consuming to use, so it will certainly not replace the Jamar in everyday clinical practice. There is an abundance of data collected with each measurement cycle, making it difficult to interpret the results. Clear weakness of the study is the purely subjective assessment of the training status of the hand and the participant himself, so that the results based on the assumptions can only be used with reservation.

## Conclusions

The Jamar-dynamometer only measures grip force globally. Big advantage of the manugraphy system is that the measuring device is based on the sum of forces distributed over the surface of a cylinder and therefore allows for differentiated measurement even with low impact forces. It can provide a good resolution for localized pathologies and offers the perspective to better understand the biomechanics of the impaired hand. Hand strength is significantly influenced by sex and hand length. These factors should be taken into account in scientific publications that include measurements of hand strength as a comparative parameter in terms of the outcome of a treatment method. What appears to be important is that standard values for grip strength cannot be established. The comparison between the injured hand and the contralateral hand is of greater importance than the comparison to a normative collective. The manugraphy system provides similarly stable values, shows the same influencing factors and is similarly robust against potential confounders as the well-established Jamar dynamometer technique. As a recently introduced method for determining grip strength with defined local resolution, the device encourages further clinical studies on changes of force distribution in special disease patterns of the hand.

## Abbreviations

cm: Centimeter
Hz: Hertz
IBM: International Business Machines
kg: kilogram
kPa: 1000 Pascal
m: meter
max: maximum
min: minimum
mm: millimeter
N: Newton
n: number of observations
PP: Personal computer
r: reliability
s: second
SD: Standard deviation
SPSS: Statistical package for the social sciences
v: validity
x: mean value
